# Pyrene: A Probe to Study Protein Conformation and Conformational Changes

**DOI:** 10.3390/molecules16097909

**Published:** 2011-09-14

**Authors:** Gursharan Bains, Arti B. Patel, Vasanthy Narayanaswami

**Affiliations:** 1 Department of Chemistry and Biochemistry, 1250 Bellflower Boulevard, California State University Long Beach, Long Beach, CA 90840, USA; 2 Children’s Hospital Oakland Research Institute, 5700 Martin Luther King Jr. Way, Oakland, CA 94609, USA

**Keywords:** pyrene, excimer, monomer, *Py* value, apolipoproteins, fluorescence, protein oligomerization, protein-lipid interactions, protein-membrane interactions

## Abstract

The review focuses on the unique spectral features of pyrene that can be utilized to investigate protein structure and conformation. Pyrene is a fluorescent probe that can be attached covalently to protein side chains, such as sulfhydryl groups. The spectral features of pyrene are exquisitely sensitive to the microenvironment of the probe: it exhibits an ensemble of monomer fluorescence emission peaks that report on the polarity of the probe microenvironment, and an additional band at longer wavelengths, the appearance of which reflects the presence of another pyrene molecule in spatial proximity (~10 Å). Its high extinction coefficient allows us to study labeled proteins in solution at physiologically relevant concentrations. The environmentally- and spatially-sensitive features of pyrene allow monitoring protein conformation, conformational changes, protein folding and unfolding, protein-protein, protein-lipid and protein-membrane interactions.

## 1. Introduction

Biochemists and biophysicists have an arsenal of fluorescent probes at their disposal to understand the molecular organization of biological molecules. This review describes the use of pyrene and its derivatives as a probe to study biomolecules. Pyrene is one of the oldest probes to be employed to study a wide range of biomolecules: Lipids, proteins and nucleic acids. The review will focus only on the use of pyrene to monitor conformation and conformational changes in proteins in aqueous solutions. A brief introduction of the salient fluorescence spectroscopic features of pyrene is provided, that may be of relevance from a biochemical perspective. The exchangeable class of apolipoproteins is used to illustrate the application of pyrene as a fluorescent probe to study protein conformation, conformational changes and dynamics. Finally, a few examples where pyrene has been used to study protein conformation are discussed. Discussion of pyrene as a probe to study lipids and nucleic acids in membrane biophysics, cell biology and cellular biochemistry is beyond the scope of this review; the reader is referred to comprehensive reviews on these topics [1,2,3,4,5,6].

## 2. Site-Specific Labeling of Proteins with Pyrene

Proteins are typically conjugated with pyrene by labeling lysines [7,8,9,10] or cysteines, using the reactivity of succinimidyl ester, isothiocyanate and sulfonyl chloride groups for the former, and maleimide and iodoacetamide groups for the latter [11]. The thiol reactivity of maleimide and iodoacetamide functional groups is used to achieve labeling under carefully controlled pH conditions ensuring that labeling occurs predominantly at cysteine residues. However, when using ‘thiol-specific’ fluorophores for labeling proteins, consideration must be given for potential attachment of these probes to other side chains and/or non-specific binding to hydrophobic sites; for example, the ε-amino group of lysine was labeled with acrylodan [12], a probe generally used to label sulfhydryl groups. Therefore, data obtained with thiol-specific labeling studies need to be interpreted with caution. Nevertheless, cysteines are the preferred target due to their lower frequency of occurrence compared to lysines. In case of proteins with naturally occurring cysteines, the probe will monitor all cysteines if stoichiometric labeling is achieved. If cysteines are not present at locations of interest, they may be replaced by serines or alanines (which show negligible reactivity with sulfhydryl reagents) provided the function of the protein is not significantly altered [13,14]; this is followed by introduction of cysteine(s) at desired site(s) by site directed mutagenesis, and labeling with pyrene.

**Figure 1:**
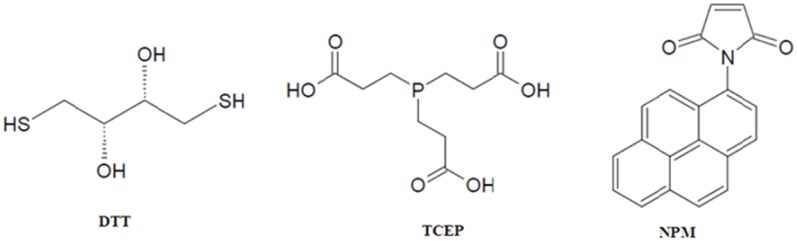
Structures of reducing agents dithiothreitol (DTT) and tris(2carboxyethyl)phosphine (TCEP), and sulfhydryl-specific fluorophore *N*-(1-pyrene)maleimide (NPM).

The labeling step with *N*-(1-pyrene) maleimide (NPM, Figure 1) is preceded by pre-incubation of proteins with freshly prepared reducing agents such as dithiothreitol (DTT, Figure 1) or tris(2-carboxyethyl)phosphine (TCEP, Figure 1) to reduce potential disulfide bonds [sometimes carried out in the presence of chemical denaturants such as guanidine-HCl (GdnHCl) to gain access to buried disulfide bonds], with a minimum of two-fold molar excess per cysteine residue. While it is a highly efficient reducing agent, the disadvantages of using DTT are its susceptibility to air oxidation and its own reactivity with the NPM that is introduced at the labeling step, which will compete with labeling sites on the protein. Following reduction, DTT may be removed by dialysis or by gel filtration prior to labeling, although there is a possibility that there may be reformation of the disulfide bonds. The practice of using ten-fold molar excess of NPM over DTT has overcome this issue in select cases [15,16,17,18]. Using TCEP at neutral pH has advantages: (i) its resistance to air oxidation; (ii) it does not contain thiol groups and therefore it does not need to be removed prior to labeling; (iii) it is non-reactive with other functional groups in proteins; (iv) it mediates rapid reduction (<30 min) at room temperature. Reduction is immediately followed by NPM labeling, sometimes in the presence of GdnHCl. Extensive dialysis, gel filtration or affinity chromatography is employed to remove excess reducing agent, NPM and GdnHCl [18]. NPM is essentially non-fluorescent in its unbound state in aqueous environments [11].

The stoichiometry of labeling is calculated using the molar extinction coefficient of pyrene (~40,000 M^−1^ cm^−1^ at 338 nm in methanol [11]). While 1:1 pyrene to cysteine stoichiometry is optimal, ratios of <1 may still be used to obtain information regarding spatial proximity and probe microenvironment [18]. Information obtained from higher ratios need to be considered with caution as there may be non-specific labeling or labeling at sites other than the desired cysteine residue(s). It is important to ensure that the overall fold and function of the protein are not significantly altered as a result of the amino acid substitution(s) and pyrene labeling. Secondary structural analysis by circular dichroism (CD) spectroscopy has been typically employed to assess potential changes in overall folding; in cases where the presence of the pyrene moiety affects CD measurements, infrared spectroscopy has been employed as a useful alternative [17].

## 3. Photophysics of Pyrene Fluorescence Emission

For pyrene-labeled proteins, two unique spectral features of the fluorescence emission spectra are exploited to obtain information regarding protein structure, molecular organization and conformation. This section is devoted to the introductory photophysical aspects of these features. For an in-depth understanding and further information regarding the photophysical processes that govern the fluorescence and absorption characteristics of pyrene in solvents and protein-bound pyrene in aqueous solutions, the reader is referred to several excellent reviews by pioneers in this field [19,20,21,22]. Section 4 and Section 5 will describe the application of these features using specific examples.

### 3.1. Py Value

The fluorescence emission spectrum of pyrene is characterized by five major vibronic bands designated Bands I, II, III, IV and V, with well defined peaks at ~375, 379, 385, 395 and 410 nm, respectively. To differentiate them from the fluorescence arising from dimer interaction (described below), the ensemble of the five vibronic bands is collectively referred to as the monomer bands. The first notable feature useful for analysis of protein conformational changes is the exquisite sensitivity of the peak at 385 nm (corresponding to the third vibronic band with 0–2 transition) to the polarity of the probe’s microenvironment, a consequence of the coupling of electronic and vibronic states [23,24,25]. In comparison to the emission intensity of Band I at 375 nm (which corresponds to the first vibronic band with 0-0 transition), the intensity of Band III at 385 nm is significantly enhanced in hydrophobic environments. In contrast, the intensity of Band I is significantly higher than that of Band III in polar environments [26,27]. The ratio of the fluorescence emission intensities of Band I/III vibronic band (Py value) can thus be employed to detect polarity in the vicinity of the probed location.

**Figure 2:**
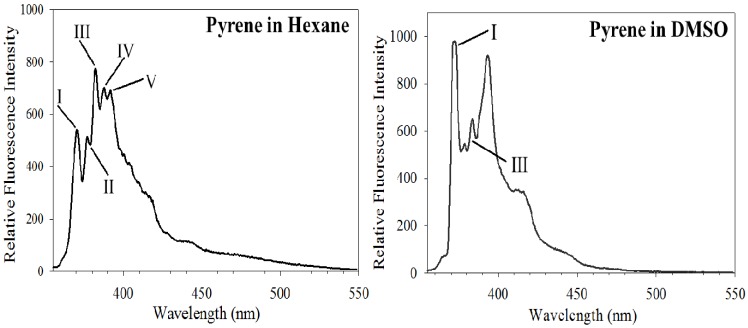
Pyrene fluorescence emission is sensitive to solvent polarity. Fluorescence emission spectra of pyrene were recorded in solvents of varying polarity such as hexane and DMSO. Arrows point to Bands I, II, III, IV and V for the spectrum in hexane. Only Bands I and III are shown for DMSO (figure reproduced from [18], with slight modifications to the legend, with permission from the American Chemical Society, © 2010).

The sensitivity and dependence of the intensity of Band III on the polarity of the microenvironment surrounding the probe are illustrated by comparing the emission spectra of free pyrene in hexane (Figure 2, Left) and dimethylsulfoxide (DMSO) (Figure 2, Right). These represent solvents with low and high dielectric constants (4.8 and 47.2, respectively) and low and high dipole moment (0 and 3.96 Debye, respectively). In hexane, pyrene displays a dominant Band III relative to Band I, while in DMSO, Band I is dominant compared to Band III. The reasons for these observations have been attributed to excited state interaction of the molecule with the surrounding solvent (H-bonding and other interactions between the solute dipole moment and that of the solvent) and solvent reorientation around the excited state dipole [28,29]. Thus, by comparing the *Py* value, it is possible to infer the polarity of the site in the vicinity of the probe [17,20,24,30,31]. Comparing the ratios in two different environments within a study is more informative than the absolute values of the ratios *per se*. This feature can be used to assess changes in probe microenvironment as a result of protein conformational change, protein/membrane interaction and protein unfolding.

### 3.2. Excimer Emission

A second notable feature of pyrene fluorescence emission that can be employed for protein conformational analysis is the appearance of a broad, unstructured band at longer wavelengths (ranging from 425 to 550 nm, centered around 460 nm) when two pyrene rings are ~10 Å from each other. It arises due to formation of an excited state dimer or ‘excimer’ and involves interaction between two pyrenes [21,32]. The unusually long lifetime of pyrene emission (>100 ns) [27,31] allows this excited state reaction to occur. The fluorescence emission spectra of pyrene-conjugates exhibiting excimer band deviate from the mirror-image rule [33]. Excimer emission may arise in a dose-dependent manner (for example at higher concentration of pyrene-labeled protein in solution or in the context of the plane of a lipid bilayer) or when two pyrenes are spatially proximal to each other in an intra-molecular context.

The monomer/excimer (*m/e*) ratio, calculated by comparing the fluorescence intensity (or quantum yield) of the first monomer peak (typically ~375 nm) with respect to the excimer band (generally ~460 nm), is a *relative* indicator of the extent of excimer formation, and therefore the spatial proximity between two pyrene moieties. As with the *Py* value, the absolute value of the *m/e* ratio yields little information per se; it is more informative to compare ratios of a given set of labeled proteins to obtain details regarding the relative proximity between specified sites. The photophysics behind pyrene fluorescence is a complex phenomenon involving multiple species and processes [20,21,34,35]. A simplistic scheme adapted from Lehrer [21] is shown below to describe selected species and processes involved in pyrene fluorescence emission for purposes related to protein conformational analysis in aqueous solutions:

M → M* → M + hν_M_  (monomer emission)

Scenario 1:

M + M → M●M → M●M* → E* → M●M + hν_E_ (excimer emission)

Scenario 2:

M + M → M●M → M●M* → M●M (static quenching; non-fluorescent)

Excitation of a ground state monomer (M) gives rise to an excited state monomer (M*), decay of which results in typical monomer emission spectrum composed of an ensemble of peaks between 375 and 410 nm (hν_M_). When M interacts with a spatially proximal M to yield M●M, two scenarios need to be considered: In Scenario 1, M●M is in a precise configuration, which upon excitation (M●M*) leads to excimer (E*) formation; excimer formation is manifest as an excimer emission peak: A broad, red-shifted band around 460 nm (hν_E_). In Scenario 2, M●M is a non-fluorescent complex that is statically quenched (*i.e.*, it is not in a favorable configuration for excimer formation). Whether two proximal pyrenes lead to excimer formation or a non-fluorescent complex appears to be determined by the local microenvironment. Analysis of the excitation and absorption spectra of pyrene-labeled proteins yields further information that aids in distinguishing between these scenarios [21,36,37,38,39].

## 4. Change in *Py* Value Reflects Protein-Membrane Interaction

This section examines a specific example of α-synuclein to demonstrate the use of pyrene Band III sensitivity to the polarity of its microenvironment in obtaining information about protein-membrane interactions. α-synuclein belongs to a family of small, soluble proteins (~140 residues) that plays a key role in Parkinson’s disease, a neurodegenerative disorder characterized by dramatic losses of dopaminergic neurons. It is composed of: (a) an N-terminal domain with seven conserved 11-residue repeats, bearing a propensity to form amphipathic α-helices and to interact with lipid surfaces [40]; (b) a beta-amyloid binding domain, and, (c) a C-terminal tail, characterized by an abundance of negatively charged residues that bind Ca^2+^ [41,42]. Under physiological conditions, α-synuclein is found in a soluble form in the cytosol in a predominantly unfolded state [43]. It is acknowledged that the pathogenesis triggered by α-synuclein is likely due to membrane-binding induced structural alterations involving transition from a monomeric unstructured state to a β-sheet conformation. However, details regarding the mechanistic basis of membrane interaction, specifically the role of Ca^2+^ on the conformation of the acidic tail was not clearly understood. We used the sensitivity of Band III intensity to its microenvironment polarity to obtain further information regarding α-synuclein/membrane interaction [17].

Recombinant α-synuclein constructs bearing a single cysteine either at position 11 or 124 (A11C or A124C) were generated to serve as sites for NPM labeling to monitor the helical N-terminal domain and the acidic C-terminal tail, respectively. Fluorescence emission spectra of the labeled variants in lipid-free state revealed well-defined vibronic bands attributed to monomeric species. Excimer emission was noted to a minor extent in pyrene-A11C-α-synuclein (but not in pyrene-A124C-α-synuclein). This was attributed to protein-protein interactions of the amphipathic α-helices in the N-terminal domain. The abundance of negatively charged residues in the C-terminal tail likely causes repulsion between individual α-synuclein molecules in lipid-free state. Upon interaction with vesicles composed of phosphatidylcholine and phosphatidic acid, three significant changes occurred: (i) a large increase in the fluorescence quantum yield for both variants; (ii) loss of the excimer emission in pyrene-A11C-α-synuclein, with no changes noted for pyrene-A124C-α-synuclein; and (iii) appearance of a more pronounced Band III peak for pyrene-A11C-α-synuclein, indicative of binding of the α-helical N-terminal domain to the lipid surface; on the other hand, pyrene-A124C-α-synuclein displayed an insignificant Band III peak in both lipid-free and lipid-bound states, indicating that the C-terminal acidic tail was not associated with the lipid surface. The N-terminal helix appears to anchor the entire molecule to the membrane, with the acidic tail remaining unstructured and unbound.

**Figure 3:**
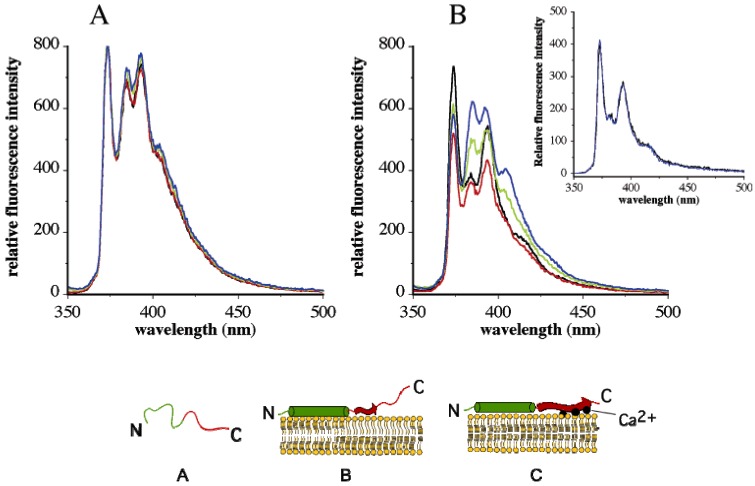
Band I/Band III ratio is an indicator of protein membrane interaction. Fluorescence emission spectra of singly labeled pyrene-A11C- (Left) and A124C-(Right)-α-synuclein in lipid-bound states were recorded in the absence and presence of varying amounts of Ca^2+^. *Inset*: Effect of 0 (black) and 1 mM Ca^2+^ (blue) on fluorescence emission characteristics of *lipid-free* pyrene-A124C-α-synuclein. *Bottom*: Model showing α-synuclein as an unstructured protein in lipid-free state N-terminal domain in green and C-terminal domain in red (**A**); upon lipid interaction, the protein is anchored via its N-terminal domain (green cylinder), with the C-terminal acidic tail remaining unbound (**B**); the presence of Ca^2+^ triggers membrane interaction of the acidic tail, possibly by forming “Ca^2+^ bridges” (C) (reproduced from [17] with permission from the American Chemical Society, © 2006).

Interestingly, the presence of Ca^2+^ appeared to have a dramatic effect on the spatial disposition of the acidic tail in lipid-bound α-synuclein. A significant increase in the intensity of Band III relative to Band I was noted for pyrene-A124C-α-synuclein: This is reflected as a decrease in the *Py* value from 1.99 in the absence of Ca^2+^ to 1.4 in the presence of 0.5 mM or higher Ca^2+^ concentrations (Figure 3A and 3B). The change in *Py* value is indicative of a more hydrophobic microenvironment in the vicinity of position 124. This suggested that the acidic tail of α-synuclein, which had hitherto remained unbound in the absence of Ca^2+^, is likely interacting with the lipid surface in its presence. The membrane apposition of the acidic tail was unique to Ca^2+^, as other divalent cations such as Mg^2+^ (or monovalent cations such as Na^+^) did not elicit a similar response. These studies indicate that the effect is Ca^2+^-dependent and not due to any nonspecific effect of the pyrene fluorophore. The presence of Ca^2+^ had no significant effect on the fluorescence emission profile of lipid-associated pyrene-A11C-α-synuclein, with the *Py* value remaining unchanged at all concentrations. Ca^2+^ did not have any effect on Band III intensity of lipid-free pyrene-A124C/α-synuclein.

Based on these observations, the authors proposed a model (Figure 3), wherein α-synuclein initially interacts with lipid surfaces via its N-terminal helix, which also serves to anchor the entire protein to the membrane surface. This is followed by a Ca^2+^-triggered membrane association of the acidic tail to the negatively charged head groups of phosphatidic acid at the surface of the membrane, *i.e.*, “Ca^2+^ bridging”. Negative charge clusters are often known to coordinate divalent ions such as Ca^2+^ [44], in conjunction with variable number of carbonyl backbone atoms and anionic head groups of phospholipids. In the case of α-synuclein, Ca^2+^ bridging may play a role in membrane apposition of the C-terminal tail, thereby triggering β-sheet formation, a potential mechanism leading to α-synuclein aggregation, an observation confirmed by CD spectroscopy. The model developed from spectroscopic analysis presents the possibility that α-synuclein is more prone to aggregation in lipid-bound state in the presence of Ca^2+^, a scenario likely to occur in synaptic vesicles in Parkinson’s disease neurons.

## 5. Excimer Emission Is an Indicator of Spatial Proximity in Proteins

### 5.1. Intra-Molecular Distance Relationships

There are numerous examples of studies where intra- and inter-molecular interactions and spatial relationships in proteins were obtained from studying pyrene excimer fluorescence. Section 5 is devoted to illustrating the use of this feature using examples from our experience with exchangeable apolipoproteins and other specific cases. Exchangeable apolipoproteins are predominantly α-helical proteins that possess an inherent structural adaptability that allows them to exist in lipid-free and lipoprotein- (or lipid-) bound states. Insect apolipoproteins such as apolipophorin III (apoLp-III) from the Sphinx moth *Manduca sexta* and *Locusta migratoria*, the migratory locust, have served as prototypes for this class of proteins [45].

**Figure 4:**
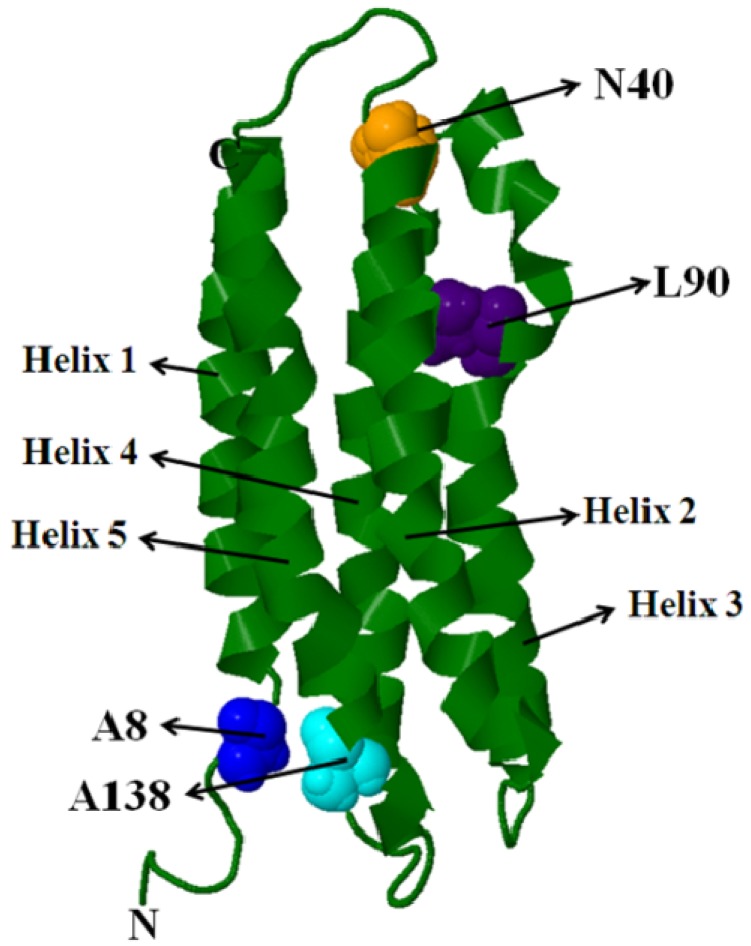
Helix bundle structure of *M. sexta* apoLp-III. The positions of substituted cysteines in the various single and double cysteine constructs used for pyrene labeling are shown as space filling model (PDB ID #1EQ1).

ApoLp-III is composed of five up-down amphipathic α-helices (numbered helix 1-helix 5; Figure 4), that are organized as a helix bundle in lipid-free state. The hydrophobic faces of the helices are oriented towards each other and the polar sides face the aqueous environment to form a stable, globular helix bundle. A remarkable feature of apoLp-III and other exchangeable apolipoproteins is their ability to convert phospholipid vesicular structures to discoidal bilayer complexes. In apoLp-III, lipid binding triggers a dramatic conformational change involving opening of the helix bundle, which allows the hydrophobic interior to interact with lipids and form a lipoprotein complex.

Based on biochemical studies of apoLp-III from *M. sexta* and *L. migratoria*, it was postulated that lipid binding induces the helix bundle to open about putative hinge loops allowing helices 1, 2 and 5 to move away from helices 3 and 4 [46,47,48]. Pyrene fluorescence analysis offered spectroscopic evidence in support of this hypothesis; further, it suggested that the lipid-triggered conformational switch involved complete opening of the helix bundle to an extended helical organization [49,50,51].

**Figure 5:**
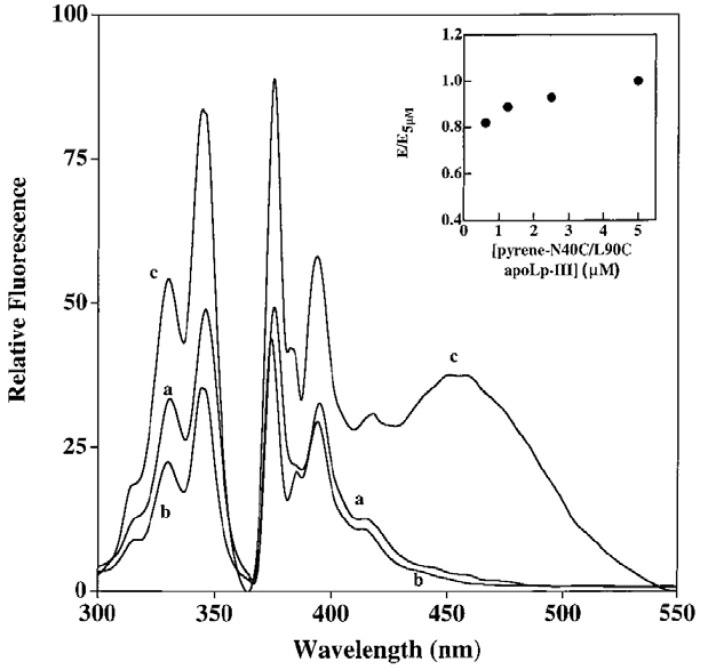
Fluorescence excitation and emission spectra of lipid-free single-labeled N40C-, L90C- and double labeled N40C/L90C-apoLpIII (*a*, *b* and *c*, respectively). Inset shows effect of “dilution” with unlabeled apoLp-III on pyrene-labeled apoLp-III excimer fluorescence. E5μM = area under excimer emission curve of a 5 μM pyrene labeled N40C/L90C-apoLp-III, and E = area at different concentrations (reproduced from [15] with permission from the American Chemical Society, © 2000).

Single cysteine residues were substituted at strategic locations on a helix being monitored: A8C, N40C, L90C and A138C on H1, H2, H3 and H5, respectively. In addition, double cysteine residues were engineered to allow pyrene labeling at positions N40C/L90C- or A8C/A138C-apoLp-III. Pyrene fluorescence emission spectra of single and double-labeled protein revealed interesting features about apoLp-III helical repositioning upon lipid interaction. The excitation and emission spectra of pyrene-labeled N40C-, L90C- and N40C/L90C-apoLpIII [15] are shown in Figure 5. Similar results were obtained with pyrene-labeled A8C-, A138C- and A8C/A138C-apoLp-III [52]. In the lipid-free state, none of the single-labeled proteins revealed an excimer band, indicating that there is no inter-molecular interactions between two helix bundles and that the proteins do not self-associate.

**Figure 6:**
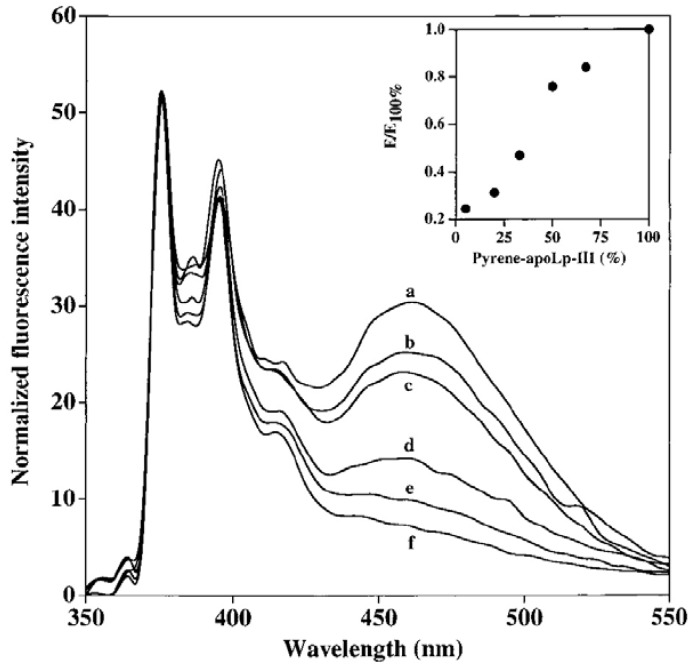
Fluorescence emission spectra of lipid-bound pyrene-N40C/L90C-apoLp-III. The spectra of lipid-bound protein with pyrene-N40C/L90C-apoLpIII only (curve a; E100%) or a mixture of labeled and unlabeled (wild type) protein where the proportion of labeled apoLp-III was 67% (curve b), 50% (curve c), 33% (curve d), 20% (curve e) and 5% (curve f). *Inset:* Effect of dilution on excimer fluorescence. E100% = area under excimer emission peak for lipid-bound complexes containing only labeled apoLp-III; E = area under excimer peak for complexes bearing the indicated percentage of labeled protein (reproduced from [15] with permission from the American Chemical Society, © 2000).

On the other hand, both double-labeled variants displayed a significant excimer emission band, which does not decrease in intensity upon mixing excess wild type unlabeled apoLp-III. This is indicative of intra-molecular spatial proximity between the specified sites; from NMR structure, the distance between the Cα atoms of N40 and L90 was 13 Å, while that A8 and A138 was 9.46 Å [50]. Upon interaction with phospholipid vesicles, disc-shaped lipoprotein complexes are obtained containing a bilayer of phospholipids surrounded by multiple molecules of apoLp-III [48]. Fluorescence emission spectra of discoidal complexes bearing pyrene-labeled-N40C/L90C-apoLp-III (Figure 6) reveal strong excimer peak, indicative of two pyrenes located ~10 Å from each other in the lipid-associated state of apoLp-III. 

**Figure 7:**
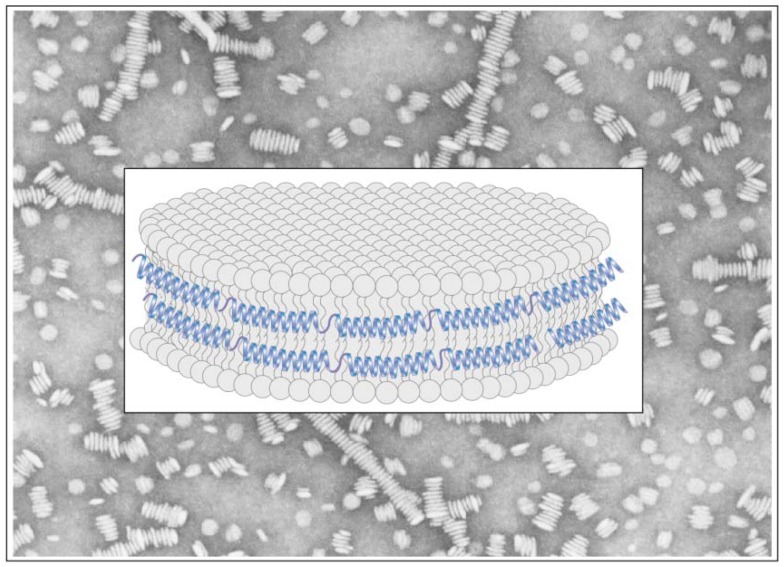
Lipid-associated conformation of *M. sexta* apoLp-III. Lipid-associated apoLp-III appears as discoidal particles (~17 nm diameter) by electron microscopy after negative staining (*background*). The discoidal structures are composed of a bilayer of phospholipids circumscribed by apoLp-III in an extended conformation, resembling a “belt” (*foreground*). Data obtained from spectroscopic studies [15,49,51,52] are consistent with a model wherein the helical axes are aligned perpendicular to the fatty acyl chains of the phospholipid bilayer.

To discriminate between intra- and inter-molecular excimer formation, the lipoprotein complexes were prepared with double-labeled protein “diluted” or mixed with increasing amounts of wild type unlabeled apoLp-III. The expectation is that on average each pyrene-labeled apoLp-III will be adjacent to an unlabeled molecule. A gradual decrease in fluorescence emission intensity at ~460 nm with increasing proportion of unlabeled protein was noted, indicating that the excimer emission was from inter-molecular proximity between the specified sites. Taken together with pyrene excimer analyses of A8C/A138C-apoLp-III, and mixtures of different singly-labeled molecules, we proposed a “belt model” of lipid-associated apoLp-III (Figure 7). The open “belt” conformation results from complete opening of the helix bundle, which circumscribe a bilayer of lipids to yield discoidal bilayer structures. This model is also supported by observations from Attenuated Total Reflectance Fourier transform infrared spectroscopy [51,52]. The fluorescence data suggest that neighboring apoLp-III molecules are aligned anti-parallel with respect to each other, with the possibility that the alignment of the helical segments may be offset by one helical segment.

### 5.2. Domain-Domain Distance Relationships

**Figure 8:**
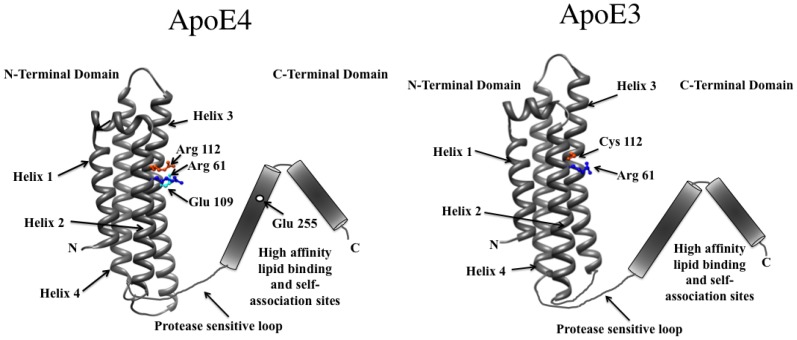
Two-domain organization of apoE3 and apoE4. The NT domains of apoE3 (Right) (PDB ID #1NFN) and apoE4 (Left) (PDB ID #1GS9) (residues 1–191) are folded into a 4-helix bundle [53,54]. The CT domain (residues 201–299) is shown as cylinders representing α-helices based on sequence predictions and biophysical analyses [60].

While comparing intra molecular interactions between two domains in mammalian apolipoprotein E (apoE), pyrene excimer fluorescence provided supporting information regarding spatial proximity between crucial sites relevant in lipid transport function. ApoE is composed of 299 residues that are folded into two domains: An N-terminal domain (residues 1–191) and a C-terminal domain (201–299) linked via a protease-sensitive loop. Three major isoforms of apoE have been identified in humans, apoE2, apoE3 and apoE4, which bear an identical sequence except at positions 112 and 158: apoE2 has a Cys, while apoE4 has Arg at both these locations; apoE3 has a Cys at 112 and Arg at 158. While apoE3 is considered an anti-atherogenic protein, apoE4 is considered a major risk factor for heart disease and Alzheimer’s disease. Understanding the structural basis of the difference between these isoforms has been rendered difficult due to the non-availability of high-resolution structural information of these proteins in their tetrameric configuration and due to lack of understanding of the precise molecular mechanisms of their roles in lipid binding. Comparison of the X-ray crystal structures of the NT domains of apoE3 and apoE4 [53,54] reveals 4 long amphipathic α-helices (labeled helix 1–helix 4) folded in an up-down manner to form a helix bundle (Figure 8) similar to the helix bundle structure of insect apoLp-III [45,55,56]. However, the presence of Arg at position 112 in apoE4 (which forms a salt bridge with Glu109) causes Arg61 (located in helix 2) to face outward. Biochemical and biophysical studies suggest Arg61 forms a salt bridge with Glu255 in the CT domain, thereby giving rise to the NT and CT domain interaction hypothesis in apoE4 [57,58]. Pyrene fluorescence analysis of double-labeled apoE4 (Arg61Cys and Glu255Cys) [16] revealed the appearance of an excimer band, providing spectroscopic support for the proposed proximity between these sites [54,57,59].

### 5.3. Excimer Emission Reveals Inter-Molecular Interactions: Protein Oligomerization, Aggregation and Polymerization

This sub-section examines various scenarios of inter-molecular interactions with specific examples of protein-protein interaction, protein oligomerization or subunit interaction, protein aggregation and polymerization using pyrene excimer fluorescence.

Lehrer and colleagues provided one of the earliest examples of analysis of protein-protein interaction using pyrene fluorescence in conjunction with other conventional approaches to contribute towards understanding the structural and conformational basis of muscle contraction [61,62,63,64]. Muscle contraction involves a sliding movement of thin filaments (composed of actin, tropomyosin and troponin) relative to thick filaments (composed primarily of myosin bearing ATPase activity), a process that is regulated by the troponin/tropomyosin complex and Ca^2+^ [65,66,67,68]. The tropomyosin-troponin complex prevents actin from stimulating ATPase activity of myosin and therefore muscle contraction at low Ca^2+^ concentrations. Our current understanding of muscle contraction emerges from studies both on striated (skeletal) and non-striated (smooth) muscles. Tropomyosin from rabbit skeletal muscles is composed of two polypeptide chains, α and β, that form an inter-molecular coiled-coil helix to yield two isoforms, αα and αβ. Both chains bear a Cys at position 190. Taken in conjunction with other studies, excimer emission of pyrene labeled tropomyosin homodimer confirmed the spatial proximity, parallel and in-register orientation of two neighboring chains [36,61,69]. The presence of pyrene lowered the mid-point of temperature-induced denaturation as observed by CD spectroscopy, indicative of localized structural perturbations; however, reasonable conclusions were made since there was minimal alteration to the overall α-helical nature of labeled-tropomyosin. A similar approach employing pyrene fluorescence was used to decipher the composition of tropomyosin from chicken gizzard smooth muscle, which is also composed of α and β chains; in this case, while the α chain bears Cys190, the β chain bears a Cys at position 36. Although the native tropomyosin is a heterodimer (displaying minimal excimer upon labeling with pyrene) [70,71], it was demonstrated that the chains have the capability to dissociate and form homodimers around physiological temperatures, as noted by the gradual increase in excimer fluorescence [70]. Pyrene located on a penultimate carboxyl terminal cysteine in a highly mobile and flexible segment of tropomyosin from a non-muscle source, also exhibited efficient excimer formation indicative of end-to-end overlap of tropomyosin molecules [72].

Further, pyrene fluorescence analysis provided a valuable tool to assess conformational changes triggered by Ca^2+^ in troponin complex and contributed towards our understanding of the interaction between tropomyosin, troponin complex and actin. For several molecules of actin, there is one each of tropomyosin and troponin complex, with tropomyosin present as a coiled-coil protein that lies alongside the muscle actin thin filament. The troponin complex is composed of three subunits, regulation by which forms the essence of muscle contraction in skeletal and cardiac tissues: Troponin C (a subunit that binds Ca^2+^), troponin I (an inhibitory subunit), and troponin T (a subunit that binds the troponin complex with tropomyosin). Ca^2+^-induced conformational changes in troponin C plays a key role in initiating muscle contraction. Troponin C (18 kDa) is composed of two globular domains or lobes at either ends of the molecule linked by a central α-helix. The central helix may be rigid and extended conferring a dumbbell-like shape to the molecule [73] or partially melted and mobile in the central part [74] bringing the two terminal domains closer to each other, depending on the isolation conditions and method of three dimensional structural determination [68]. Labeling of bovine cardiac troponin C with pyrene at the two cysteines at position 35 and 84, revealed significant excimer fluorescence, indicative of spatial proximity between these residues [75,76,77]. Binding of Ca^2+^ to the high affinity sites in the C-terminal domain had little effect on the excimer fluorescence; however, binding to the low affinity regulatory site at the N-terminal domain triggered a decrease in excimer fluorescence, implying movement of the proximal cysteines away from each other. Similar conformational reorganization was noted upon incorporation of troponin C into the troponin complex [77,78].

Troponin I is a 20 kDa inhibitory subunit of troponin that interacts with troponin C and with actin and keeps the actin-tropomyosin complex in place [79]. The presence of troponin I prevents myosin from interacting with actin in the relaxed state, *i.e.*, it inhibits actomyosin-ATPase activity at low Ca^2+^ concentrations. During muscle contraction, Ca^2+^ binding to troponin C causes a conformational change that dissociates troponin I from troponin C; this leads to tropomyosin vacating the binding site on actin for myosin, leading to muscle contraction, *i.e.*, the inhibitory activity of troponin I is abolished in this state in the presence of Ca^2+^. An N-terminal segment of Troponin I interacts with troponin C and stabilizes the interaction via multiple points of contact involving both lobes and the central helix [80,81].

One of the experimental approaches that suggested conveyance of Ca^2+^-mediated changes in troponin C conformation to troponin I and troponin T was fluorescence analysis of pyrene-labeled troponin I from rabbit and chicken skeletal muscle [79,82]. Troponin I from both sources contain two cysteines at positions 48 and 64, which were labeled with pyrene. However, that from rabbit skeletal muscle has an additional cysteine at position 133, which is the only accessible cysteine when troponin I is present as a complex with troponin C and troponin T [83]. The authors take advantage of its accessibility to block cysteine 133 with iodoacetamide in native troponin, followed by isolation of troponin I from the complex, and pyrene labeling of isolated troponin I under denaturing conditions [79]. The fluorescence emission spectra of pyrene-labeled troponin I reveals excimer bands, indicative of spatial proximity of positions 48 and 64. Upon complex formation with troponin C, no changes were noted in the absence of Ca^2+^. However, in the presence of Ca^2+^ bound to the low-affinity site in the N-terminal lobe of troponin C, a substantial decrease in excimer emission was observed indicative of further distance separation between the labeled sites. Since other studies had suggested that these cysteines are involved in binding to troponin T [83] the authors proposed that Ca^2+^-induced conformational changes in troponin C are transmitted to troponin T via troponin I.

Subsequent availability of the high resolution structure of human cardiac troponin complex in Ca^2+^-saturated form confirmed the extensive intermolecular contacts between the subunits [84]. It further confirmed the possibility that Ca^2+^ binding to the regulatory site of troponin C triggers a series of conformational changes that eventually leads to muscle contraction. Similar fluorescence analysis of chicken troponin I using pyrene excimer [82] revealed that conformational changes in troponin I may be essential for the overall process of muscle contraction.

Pyrene maleimide- [63] or pyrene iodoacetamide- [64] labeled tropomyosin was used to study interactions between tropomyosin and actin and/or myosin sub-fragment 1 [85]. The presence of pyrene maleimide at Cys190 in tropomyosin caused a 10% decrease in α-helical content, an increase in the extent of localized unfolding and a decrease in the pretransition temperature in heat-induced unfolding studies; the perturbation appeared to occur to a lesser extent with pyrene iodoacetamide. Nevertheless, their data suggest that actin and the myosin sub-fragment 1 induce significant changes in: The microenvironment of cysteine 190, chain-chain interactions in tropomyosin, and the interaction between actin and tropomyosin [64]. In addition to information on conformation, pyrene-labeled tropomyosin was recently used to monitor the kinetics of transition of actin between the inactive, intermediate and active states in the actin-tropomyosin-troponin complex since the position of tropomyosin on actin regulates the activity of the actin to stimulate ATP hydrolysis by myosin [86]. A two-step model was proposed to explain the transition from the active state of regulated actin to the inactive state. In the absence of Ca^2+^, the inactive state was predominant, while at saturating levels of Ca^2+^, the intermediate state was dominant, but with the significant presence of both the inactive and active states. Similar approaches were employed to study the effect of N-terminal acetylation of tropomyosin on interaction with actin [87], the role of Ca^2+^-mediated regulation of tropomyosin by Ca^2+^-binding proteins such as calcyclin [88], caldesmon and caldmodulin [89].

Another example of inter-molecular interaction is provided by studies involving the CT domain of apoE (residues 201–299; Figure 8). Bearing high affinity lipid binding sites, this domain mediates apoE tetramerization via helix-helix interactions between subunits. *In silico* structural predictions suggest that residues 210-266 form a long class A amphipathic α-helix and residues 268–289 form a class G* helix [90,91]. The distinguishing feature of class A helices is the clustering of positively charged residues at polar/non-polar interface and negatively charged residues at the center of the polar face. Class G* helices lack clustering of charged residues, and have a random distribution of negative and positive residues around the perimeter of the polar face. Class G* helical segments are found to be involved in protein-protein interaction and subunit formation [91,92]. Truncation analysis [93,94] and targeted substitution of bulky residues [95,96] in the CT domain suggest that the terminal helix is involved in apoE tetramerization in the absence of lipids.

**Figure 9:**
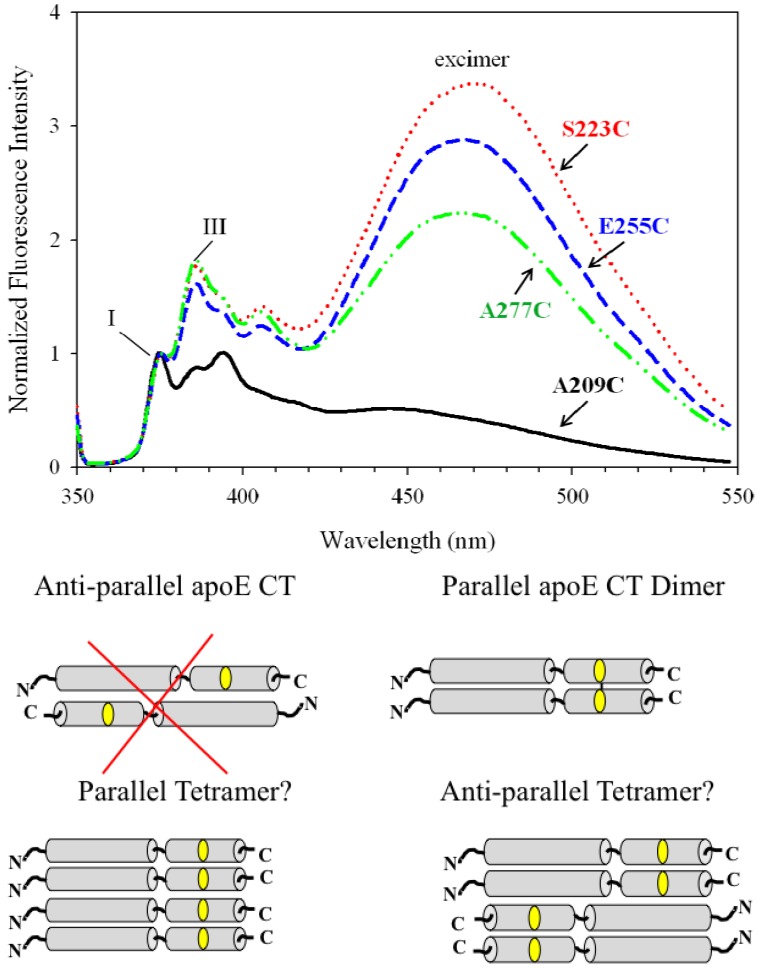
Fluorescence emission spectra of pyrene labeled apoE CT variants report on spatial proximity and relative chain orientation. *Top*: Fluorescence emission spectra of 5 μg/mL apoE CT labeled with pyrene at positions 209 (black), 223 (red), 255 (blue) and 277 (green). Band I and Band III at 375 and 386, respectively, are also shown. *Bottom*: Model showing the entire C-terminal domain of apoE making extensive inter-molecular helix-helix contact to form a dimer; anti-parallel orientation is excluded since excimer fluorescence was noted regardless of the probe location. The dimer dimerizes further to form a parallel or anti-parallel tetramer (which cannot be distinguished based on the available data) (reproduced with slight modifications from [18], with permission from the American Chemical Society, © 2010).

Our studies indicated that a larger segment including residues predicted to form the class A helix may be involved in protein-protein interaction via inter molecular coiled-coil helix formation leading to dimerization of apoE CT domain [60], and that two dimers may dimerize further to form a tetramer. Site-directed fluorescence labeling of isolated apoE CT domain (residues 201–299) with pyrene probing different locations of the class A helical segment (positions 223 or 255) and the class G* segment (position 277) yielded new information supporting this molecular organization. It demonstrated that pyrene exhibits excimer emission regardless of its location on the different predicted helical segments of apoE CT [18] (Figure 9, *Top*). Interestingly, the excimer bands in these variants displayed very high intensities relative to the monomer peak at 375 nm (*m/e* ~0.3). A pronounced Band III peak for pyrene located at positions 223, 255 and 277 (*Py* value ~0.4) indicated that these sections are located in a hydrophobic environment such as that encountered at the helix-helix interface. Thus both the class A and class G* helices in apoE CT appear to align parallel with corresponding segments in a neighboring molecule to form a dimer (Figure 9, *Bottom*). Although the pyrene fluorescence data did not offer information regarding further self-association of apoE CT dimer, it appears that the dimers dimerize further to form a tetramer. The fluorescence data available so far does not allow us to distinguish between the two possible orientations of the dimers in the tetrameric configuration. Overall, the data indicate the entire CT domain of one molecule is in intimate contact with a neighboring molecule establishing extensive inter-molecular helix-helix interactions in the lipid-free state.

A nagging question that arises when interpreting pyrene excimer emission in proteins is: Does the tendency of the hydrophobic aromatic pyrene rings to stack drive the juxtaposition of segments of protein? This issue was addressed [18] by placing pyrene at a location (A209C) in apoE that was predicted to lie outside the helical segment in apoE CT. A dramatically lowered excimer (Figure 9) and a *Py* value > 1.0 (reflecting a polar microenvironment), indicate that the aromatic stacking interaction of pyrene rings from neighboring molecules may not be the sole driving force for helix-helix spatial proximity in apoE CT. Nevertheless, it would be prudent to include appropriate controls in a given system to exclude this possibility with other proteins.

In other studies, investigators monitored the appearance of pyrene excimer to understand the supra-molecular structure of microsomal glutathione (GSH) S-transferase formed as a result of protein aggregation [97]. The enzyme is composed of four polypeptide chains with an apparent molecular weight of ~17,000, and is activated by sulfhydryl reagents; sedimentation equilibrium ultracentrifugation studies revealed that a complex of the enzyme and Triton X-100 retains enzymatic activity and has a molecular weight of ~127,000, suggesting that it exists as a functional trimeric complex [98,99]. The authors noted a concentration-dependent increase in enzyme activity that was accompanied by an increase in excimer intensity in pyrene-labeled GSH S-transferase. They proposed a model that involves aggregation of the trimeric complexes to a hexameric form, and suggested that at *in vivo* concentrations (~1 mM), microsomal GSH S-transferase may be present in an aggregated state in the endoplasmic reticulum.

Earlier studies employed pyrene-labeled actin (single labeled wild type actin or double labeled) to obtain insights into the complex interaction between actin-tropomyosin-myosin sub fragment 1 in reconstituted thin filaments or actin polymerization [63,64,88,100,101,102,103,104,105,106] and to monitor the effect of caldesmon on actin polymerization [107]. Examining the role of a specific loop region (262–274) in actin on filament formation and stabilization, Feng *et al.* [108] followed changes in pyrene excimer fluorescence emission, and established the orientation of monomers in actin filament. Monomeric actin (referred to as G-actin for globular actin) associates to form the polymeric F-actin (for filamentous actin), a process involving multiple protein machinery. These authors noted that the loop region between subdomains 3 and 4 of actin interact directly with the C-terminal domain of a neighboring molecule. Further, use of pyrene-labeled actin revealed the role of yeast formin [109] profilin [110], porin [111] and vinculin tail [112] in the turnover, restructuring and dynamics of actin polymerization and filament formation in cell cytoskeleton. Vinculin is one of the cytoskeletal proteins involved in the polymerization process; it plays a role in anchoring F-actin to the membrane. Addition of vinculin tail, also a five-helix bundle similar in structure to exchangeable apolipoproteins [113], to pyrene-labeled S265C G-actin from yeast promoted nucleation and polymerization of G-actin to F-actin bundles under physiologically relevant conditions, as deduced by excimer formation. In addition, the authors note that vinculin tail can potentially modify and remodel the organization of preformed pyrene-labeled S265C F-actin filaments.

### 5.4. Pyrene Fluorescence to Decipher Transmembrane Organization and Protein Dynamics

Examining *E. coli* lactose (lac) permease, early studies employed pyrene excimer fluorescence to understand the organization, relative spatial disposition and the dynamics of the multiple transmembrane helices [114,115]. Lac permease is a membrane transport protein involved in transduction of free energy of an electrochemical proton gradient into substrate concentration gradient to drive the uphill accumulation of β-galactosides. It is a two-domain protein with an N-terminal and a C-terminal domain, each comprised of 6 transmembrane helices (numbered helix I to helix XII) that traverse the membrane in an up-down manner. The N- and C-terminal ends of the protein are located in the cytoplasm. Site-directed fluorescence spectroscopy with pyrene provided a map of proximity relationships between the different helices in striking detail in recombinant lac permease reconstituted into proteoliposomes, as verified a decade later by solving the crystal structure of this protein at 3.5Å resolution in the absence and presence of bound ligand, β-d-galactopyranosyl-l-thio-β-d-galactopyranoside, a lactose homolog [116]. Based on the presence of excimer bands, the authors proposed that helix VIII (Glu269) is spatially proximal to helix X (His322), and helix IX (Arg302) is proximal to helix X (Glu325). The appearance of excimer emission in a double cysteine variant with pyrenes located four residues apart (His322/Glu325) also suggested that the transmembrane domain bearing these residues was in an α-helical configuration. Further, the excimer emission detected between helix VIII and helix X was quenched by water-soluble quenchers [115], an effect that was significantly decreased in the presence of β-galactosides, indicating gross tertiary structural rearrangements induced by ligands. These studies illustrate the use of pyrene fluorescence in conjunction with biochemical and other spectroscopic data to obtain information about transmembrane proteins.

Pyrene fluorescence analysis made important contributions in deciphering the transmembrane pore-forming tendency of staphylococcal α-toxin, a beta barrel protein [117]. A heptamer, α-toxin is composed of seven identical subunits of about 300 amino acids each that assemble as a transmembrane pore in the infected cell membrane causing massive changes in cell permeability. The monomers assemble into a heptameric pre-pore organization prior to formation of a transmembrane pore. Pore formation involves re-location of a centrally located 15-residue stretch towards the lipid bilayer. In addition, the N-terminal end of the protein, although not directly involved in pore formation, was implicated in the pre-pore to pore transition. Pyrene labeling of selected residues towards the N-terminal end confirmed that pore formation is accompanied by massive conformational changes involving movement of the N-terminal end in closer proximity to corresponding sites on neighboring molecules in the heptameric arrangement. This conclusion was also based on parallel experiments with polarity-sensitive probe acrylodan that reveals significant changes in the microenvironment of the N-terminal end of the α-toxin, with assembly of the heptamer prior to transmembrane pore formation.

### 5.5. Protein Unfolding

Pyrene excimer fluorescence has also been employed to monitor protein unfolding, as demonstrated with human carbonic anhydrase II (HCAII), an enzyme containing a zinc ion coordinated to three histidines in its active site [118]. High-resolution structure of HCAII reveals 10 β-strands that form an open β-sheet [119,120]; the strands are linked by loops and α-helices. Unfolding studies using chemical denaturants suggested that β-strands 3–5 form a compact residual structure, as single amino acid residues in these sites are not accessible to alkylation until high GdnHCl concentrations were achieved. This was confirmed by engineering a cysteine at position 67 in β-strand 3, and using the single naturally occurring cysteine in HCAII at position 206 in β-strand 7 to label with pyrene. The presence of significant excimer emission at concentrations of GdnHCl that caused unfolding of the overall secondary structure (as monitored by CD spectroscopy) suggested retention of a compact residual domain. Residual structures are believed to act as ‘seeds’ for protein folding [118,121,122]. In this case, pyrene excimer fluorescence offered complementary information to biochemical and other biophysical analyses to suggest that the hydrophobic interior of the protein may contribute to the residual structure of HCAII.

## 6. Advantages and Limitations of Using Pyrene

A major advantage of using pyrene for conformational analysis is its high extinction coefficient, which requires minimal amounts of protein for study. Pyrene-labeled proteins can be analyzed at very low (5–10 μg/mL), physiologically relevant protein concentrations. Its unique spectral features are ideal for studying protein-protein interactions under various conditions such as subunit interaction and oligomerization, aggregation and misfolding, and polymerization. However, care must be taken to ensure that the presence of pyrene does not affect the overall fold of the protein. Further, pyrene-labeled proteins can be used with native samples (for example, to study interaction of pyrene-labeled apoE with lipoproteins isolated from plasma) with minimal interference from intrinsic fluorescence of proteins [16], except when used as an acceptor for fluorescence resonance energy transfer.

The unique reactivity of cysteines, and the fact that they occur less frequently in proteins have been advantageous for fluorescence spectroscopic analysis in general, since one can exploit the reactivity of maleimide or iodoacetamide functional groups with –SH for labeling. However, the presence of multiple cysteines in a protein is likely to pose problems. In situations where one or two cysteines are present (but are not directly involved in function), the problem may be circumvented by substituting a serine and introducing single or double cysteines in a segment under consideration. If a naturally occurring cysteine is involved in a structural or functional role that must be preserved during analysis, labeling using the maleimide or iodoacetamide functional group presents further complications. Although pyrene isothiocyanates may be used to label the primary amino group at the N-terminal ends of proteins under carefully controlled pH conditions (~pH 7.0) [11] the possibility that labeling at the ε-amino groups of lysine side chains may occur must be seriously considered. Targeting of lysine side chains can be achieved by performing the labeling at pH 8.5–9.5.

A control to assess possible non-specific interaction of pyrene is to perform labeling reactions with wild type protein bearing no cysteine residues under conditions identical to those employed for labeling cysteine residues. The contribution to the fluorescence emission may then be subtracted from that of the labeled sample; while this may be sufficient to account for non-specific labeling, the possibility that the presence of pyrene covalently attached to cysteine(s) may alter the overall protein conformation and therefore, the extent of non-specific labeling, cannot be eliminated. Lastly, labeling with pyrene introduces a bulky group, which may not be desirable in specified cases, since the stacking of the aromatic ring systems may drive protein-protein interaction. As discussed in Section 5.3, although we cannot completely exclude it, we propose that the presence of a single pyrene molecule does not contribute significantly to apoE dimerization and excimer formation. This requires to be established in each case, making sure that the presence of pyrene does not significantly alter the secondary structure and the overall fold of the molecule, as noted in select cases [21].

As with other fluorophores, pyrene fluorescence can be quenched significantly by molecular oxygen given its long excited state lifetime [123], making it important to ensure minimal changes in oxygen concentration during sample preparation, and to carefully maintain the temperature during measurements [2].

## 7. Summary and Perspectives

Pyrene is one of the most well characterized probes in the fluorophores toolbox that has been used to study proteins, lipids, nucleic acids and other biomolecules. This review has explored the use of pyrene as a potential fluorophore to probe protein conformation using specific examples for illustrative purposes (it was not meant to cite all examples of pyrene-labeled proteins). It can be effectively used to study various scenarios such as intra-molecular (including intra domain and inter-domain) proximity relationships, inter-molecular interactions such as protein oligomerization, aggregation and polymerization, and to examine transmembrane organization, protein dynamics and folding/unfolding processes. It will be a challenging task to see if pyrene fluorescence can be exploited further to study proteins in the complex biological systems. Similar to the approach employed with aptamers, application of a combination of wavelength switching between monomer and excimer pyrenes that serve as molecular beacons and time-resolved fluorescence measurements [124,125] offers exciting future possibilities to monitor conformational changes in protein in native cellular environment.
